# Supplementary material for: Predicting visual function by interpreting a neuronal wiring diagram
Source: Nature. 2024 Oct 2;634(8032):113–23. doi: 10.1038/s41586-024-07953-5 (PMC11446822; doi:10.1038/s41586-024-07953-5)
Supplement: Supplementary file 6 — CRF and ERF predictions for individual TmY4 and TmY9 cells. Analogous to Supplementary Data 3, but for TmY target types. Shown are the top four monosynaptic pathways, the strongest pathway passing through each of the top ten intermediary types (ranking from Extended Data Fig. 7), and the trisynaptic pathway Tm1–TmY–Dm3–TmY (see the section entitled Prediction of spatial normalization). [file 41586_2024_7953_MOESM6_ESM.zip › DataS4/TmY9qΓèÑ/720575940631297384.pdf]

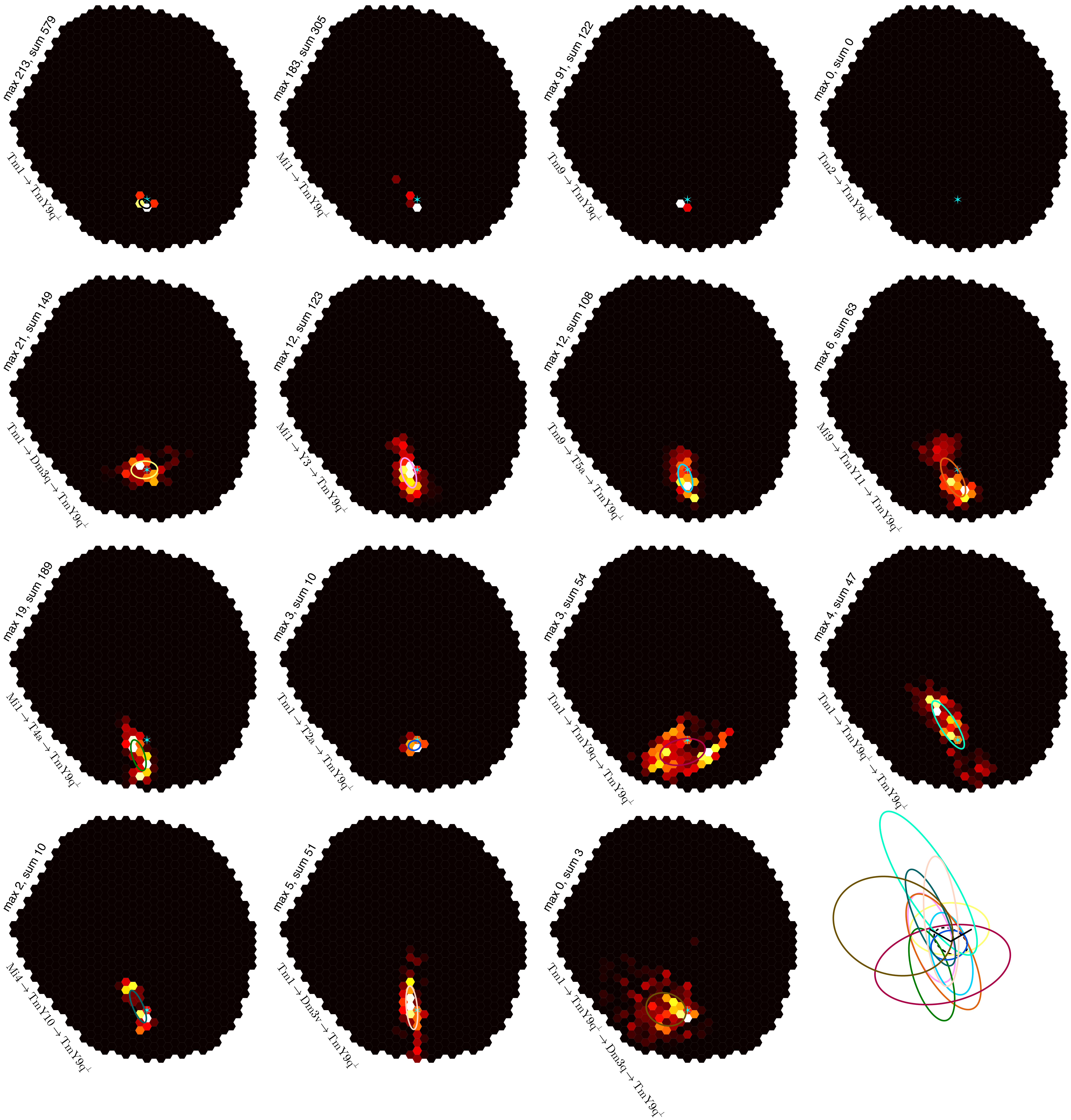
max 213, sum 579
Tm1 → TmY9q⊥
max 183, sum 305
Mi1 → TmY9q⊥
max 91, sum 122
Tm9 → TmY9q⊥
max 0, sum 0
Tm2 → TmY9q⊥
max 21, sum 149
Tm1 → Dm3q → TmY9q⊥
max 12, sum 123
Mi1 → Y3 → TmY9q⊥
max 12, sum 108
Tm9 → T5a → TmY9q⊥
max 6, sum 63
Mi9 → TmY11 → TmY9q⊥
max 19, sum 189
Mi1 → T4a → TmY9q⊥
max 3, sum 10
Tm1 → T2a → TmY9q⊥
max 3, sum 54
Tm1 → TmY9q → TmY9q⊥
max 4, sum 47
Tm1 → TmY9q⊥ → TmY9q⊥
max 2, sum 10
Mi4 → TmY10 → TmY9q⊥
max 5, sum 51
Tm1 → Dm3v → TmY9q⊥
max 0, sum 3
Tm1 → TmY9q⊥ → Dm3q → TmY9q⊥
